# First results of a uniform regional treatment protocol and registration for acute prosthetic join infection in the South-East of the Netherlands

**DOI:** 10.7150/jbji.33039

**Published:** 2019-05-21

**Authors:** Maud C. Kamp, Robin W.T.M. van Kempen, Loes Janssen, M.C. (Marieke) van der Steen

**Affiliations:** 1 Department of Orthopaedic Surgery, Catharina Hospital Eindhoven, Postbus 1350, 5602 ZA Eindhoven, The Netherlands.; 2 Department of Orthopedic Surgery, Radboudumc , Postbus 9101, 6500 HB Nijmegen, the Netherlands.; 3 Department of Orthopedic Surgery, VieCuri Medical Centre, Postbus 1926, 5900 BX Venlo, The Netherlands; 4 Orthopaedic Center Máxima, Máxima Medical Center, Postbus 90052, 5600 PD Eindhoven, The Netherlands.

**Keywords:** acute prosthetic join infection, debridement, antibiotics, irrigation, retention

## Abstract

*Introduction:* Prosthetic joint infection (PJI) is a relatively uncommon (average incidence 0.5-2%) but devastating complication, with significant morbidity and leading to tremendously increased health care costs. In 2013, delegates from nine hospitals covering a large region in the South-East Netherlands composed one combined treatment protocol for acute PJI of total hip and knee arthroplasty (THA and TKA). This protocol was based on the definition of acute PJI according to Workgroup of the American Musculoskeletal Infection Society (MSIS) and the principles of debridement, antibiotics, irrigation and retention (DAIR).

*Methods:* Patients with a THA or TKA treated with DAIR because of suspicion of PJI were selected from the online PJI database. PJI was defined as at least two phenotypically identical pathogens, isolated in cultures from at least two separate tissues, obtained from the affected prosthetic joint. Acute PJIs, occurring within 90 days after primary implantation, between January 2014 and December 2016, were analyzed. We analyzed the PJI incidence rate, patient clinical and microbiological characteristics of PJI, outcome of the DAIR treatment and adherence to the regional protocol.

*Results:* A total of 310 primary THA or TKA with a suspected PJI were registered in the regional PJI database, 236 met the definition of acute PJI, representing overall incidence of 1.12%. Following the regional treatment protocol replacement of exchangeable parts took place in 45% in 2014 to 70% in 2016. After 12 months follow-up, prosthesis retention was achieved in 87% and 3% of the patients died within one year after the primary surgery.

*Conclusion:* Results of the regional cohort are in line with the available literature. Regional collaboration and regular feedback on registered data resulted in better adherence to the combined treatment protocol. Despite our attempts to improve PJI care, PJI remains a serious complication of THA and TKA with a significant mortality rate and burden for the patient.

## Introduction

Prosthetic joint infection (PJI) is a relatively uncommon but devastating complication following total joint arthroplasty. The average reported incidence of PJI is 0.5-2%. 1-3 PJI has a large negative impact on the patient and it causes significant morbidity, mortality, and leads to tremendously increased health care costs. 1,4-6 The Workgroup of the American Musculoskeletal Infection Society (MSIS) (2011) classified PJI in 3 groups, based on duration of symptoms and time after surgery. Acute infections manifest within 3 months after primary arthroplasty. Delayed (low-grade) infections usually become manifest 3-14 months after primary arthroplasty. Late (hematogenous) infections become manifest more than 2 years after primary arthroplasty. 1,2,7 Nowadays, debridement, antibiotics, irrigation and retention (DAIR) is the most widely performed treatment for acute PJI. 3,4,6 Success after DAIR treatment can be defined as eradication of infection with retention of the prosthesis and no occurrence of PJI-related mortality. A wide range of success rates (26-95%) associated with patients who undergo debridement with implant retention has been reported in the literature. 3,4,6,8,9 Staphylococcus aureus (12-23%) and coagulase-negative staphylococci (30-43%) are the most commonly cultured microorganisms associated with acute PJI, followed by mixed flora (10-11%), streptococci (9-10%), gram-negative bacilli (3-6%), enterococci (3-7%), and anaerobes (2-4%). 1,3,10 Several attempts have been made to create treatment protocols for acute PJI. [e.g. 1,2,11,12] However, a recent survey showed that in approximately 25-30% of the Dutch Hospitals there is no protocol available for the treatment of cases suspected of acute PJI. 13 Furthermore, a lot of variety exists in the available protocols. 13,14 As national (Dutch) registries are not developed for the registration of PJI but rather focus on implant survival, not all DAIR procedures -even if exchange of modular components took place- are reported in these registries. 13,15 This contributes to the underestimation of the rate of implant-related infections 16,17.

In 2013, delegates from nine hospitals in the South-East Netherlands composed one combined diagnostic and treatment protocol for acute PJI based on the definition of acute PJI, according to MSIS and the principles of DAIR. With the aim to diminish unwanted variation and improve the quality of care around acute PJI of hip and knee arthroplasty. In order to evaluate the adherence to the treatment protocol and treatment outcome, the participating centers registered relevant aspects of treatment and outcome in a specially developed online registration system.

The purpose of this study was to give an overview of the first findings of this regional collaboration. We focus on PJI incidence rate, patient, clinical and microbiological characteristics of PJI, and outcome of the DAIR treatment. Furthermore, we evaluate the adherence to the regional protocol and describe our initiatives on how to improve this adherence.

## Methods

### Regional treatment protocol and infection database

**Figure 1:**
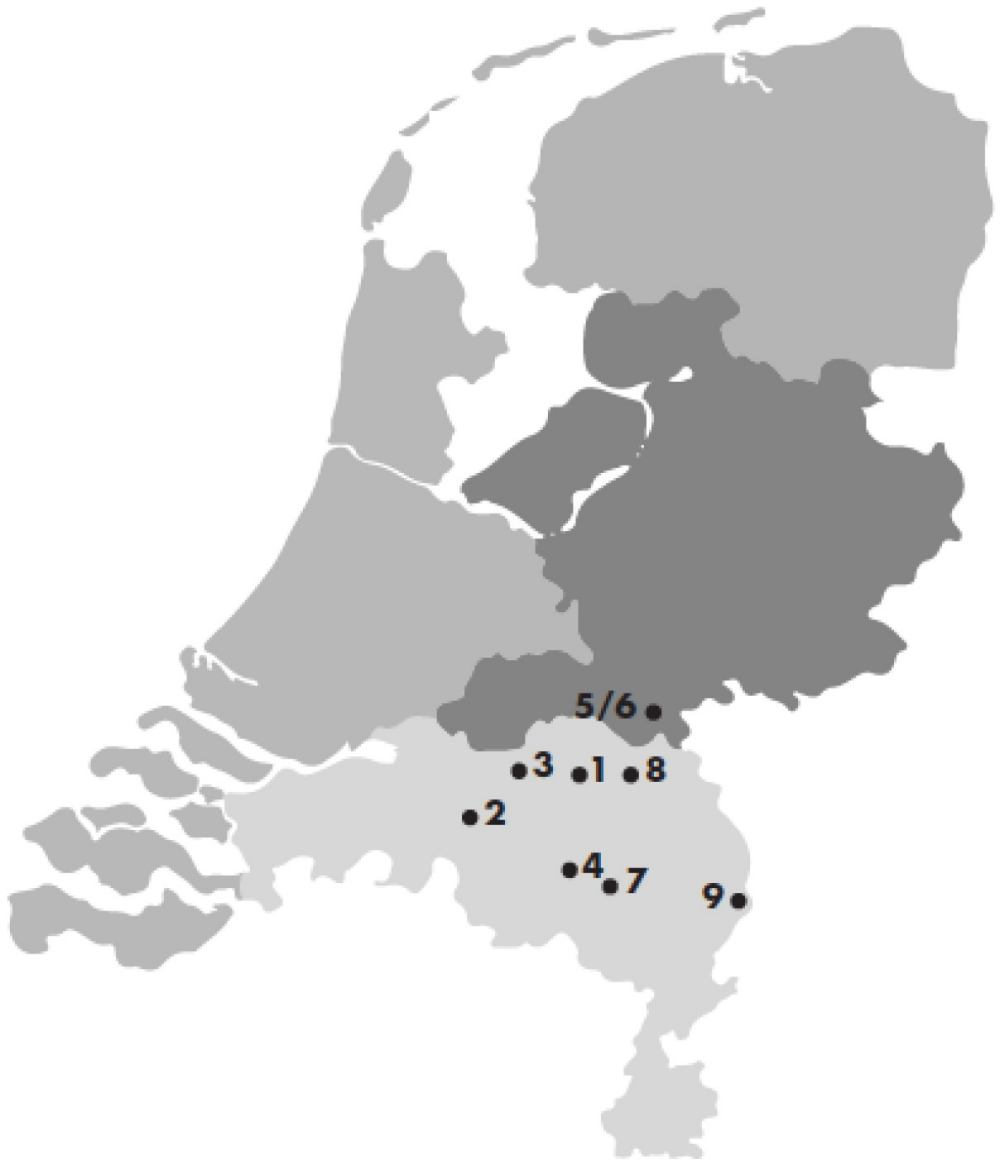
Regional infection cohort departments South-East Netherlands.

In 2013, delegates from nine orthopedic departments of hospitals in the South-East Netherlands composed one combined diagnostic and treatment protocol for acute PJI. **Figure 1.** The applied definition of PJI follows the MSIS criteria for PJI. 2,4,11,18 A PJI is present in case of a sinus tract communicating with the prosthesis. Or if a pathogen is isolated by culture from at least two separate tissue or fluid samples obtained from the affected prosthetic joint. Or if at least four of the minor criteria are present. Minor criteria are elevated serum erythrocyte sedimentation rate (ESR) and serum C-reactive protein (CRP) concentration; elevated synovial leukocyte count; elevated synovial polymorphonuclear neutrophil percentage (PMN%); presence of purulence in the affected joint; isolation of microorganism in one culture or periprosthetic tissue or fluid; and more than five neutrophils per high-power field at x 400 magnification. 2,11,18 Within the regional treatment protocol early infections are defined as presentation of PJI within 3 months after placement of the prosthesis or as a result of a hematogenous seeding.

According to the regional treatment protocol all (acute) PJIs were treated following the regimen of DAIR. The procedure consists of incising the old scar or wound, obtaining tissue samples for multiple cultures (at least 6) from synovium, capsule, and interface. This is followed by the removal of old stiches and exchangeable components (e.g. polyethylene insert) of the prosthesis. Meticulous debridement and thorough irrigation by means of pulse lavage with at least 6L NaCL is performed before exchangeable components are replaced. 1,3,6 In case of a high purulent infection at first debridement leaving behind gentamicin beads may be considered**.** Debridement and irrigation may be performed twice in the attempt to eradicate the PJI. Postoperatively, antibiotic treatment is based on bacterial susceptibility and determined in consultation with the medical microbiologist 8.

Patients with a total hip arthroplasty (THA), unipolar hip hemiarthroplasty, total knee arthroplasty (TKA) or revision THA/TKA, treated following DAIR are included in the regional PJI database. Data are acquired from the patients' electronic medical records and prospectively collected in the online regional PJI database. The data registered by each hospital in the online database include: specific operation information (e.g. type of prosthesis, operation side, operation technique), patient characteristics (e.g. sex, age, body mass index), risk factors (e.g. diabetes mellitus, malignancy, rheumatoid arthritis, smoking), clinical features (e.g. erythema, persistent wound leakage / dehiscence), date of debridement, cultured microorganisms and outcome after 3 and 12 months.

Each participating hospital has one or two contact persons (researcher / orthopedic surgeon) who is responsible for data registration, who can be contacted in case of questions by fellow surgeons and who participates in regular meetings with the other delegates. During these meetings uncertainties within the treatment protocol, challenging PJI cases and results of the registered data are discussed.

### Study design

An analysis of prospectively registered data on patients diagnosed with an acute THA or TKA PJI, between 2014 and 2016, in nine hospitals in the South-East of the Netherlands was performed. **Figure 1.** The nine hospitals comprises a variety of academic, peripheral and private clinics. Data entry was reviewed (M.K., M.vd.S.) and controversies or contradictions were double-checked and if necessary corrected by the contact person at each hospital.

### Participants

For the current analyses we included acute PJI, classified as appearance of the first signs and symptoms of infection within 90 days after primary implantation between January 2014 and December 2016. 1,2 In addition, we excluded cases of revision arthroplasty, unipolar hip hemiarthroplasty, delayed or late infection (first signs and symptoms of infection >90 days after implantation) and cases were less than two perioperative tissue cultures were obtained to analyze.

### Outcome measures

Patient characteristics, type of surgery and microbiology cultures were evaluated. Furthermore, we looked at mortality and treatment success. Treatment failure was defined as any further surgical procedure e.g. one/two-stage revision or Girdlestone situation at a follow-up of one year. Adherence to the protocol was investigated by analyzing the following key components; exchange of modular components (yes), number of cultures taken (≥6) and number of DAIR treatments performed (≤2).

### Statistical analysis

The incidence was defined as the number of prosthetic joint infections between January 2014 and December 2016 in the South-East Netherlands per 100 primary total arthroplasty procedures. This was calculated as the ratio between the number of prosthetic joint infections from January 2014 to December 2016 and the number of primary total arthroplasties in the same period. Descriptive statistics were used to describe data in terms of incidence, totals and outcome. All continuous variables are reported as means and standard deviations, categorical data as totals and percentage.

Difference over the years in adherence to the protocol and outcome were tested by means of χ2 tests. Statistical analyses were performed using SPSS (version 21.0 RES Workspace Manager statistical software).

## Results

### General

**Figure 2:**
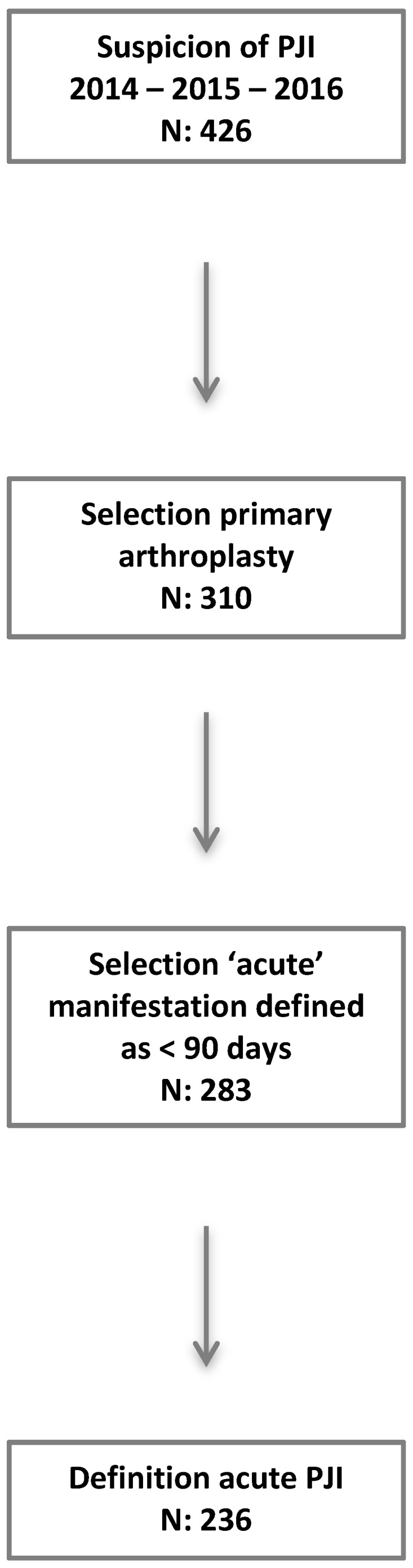
Flow chart patient inclusion

Based on data from the Dutch Arthroplasty Register, 23.353 (approximately 14% of all primary hip or knee arthroplasty in the Netherlands) 19 prosthetic hip or knee replacement procedures were performed between January 2014 and December 2016 in the nine participating hospitals. In the regional database a total of 426 suspicions of prosthetic joint infection were registered. We excluded 116 cases because of revision arthroplasty or unipolar prosthesis, leaving 310 primary TKA and THA with a suspected PJI. A total of 236 cases met the definition of acute PJI, representing an overall incidence of 1.12 %. **Figure 2.**

### Patient characteristics

Patient demographics of acute PJI and prosthetic characteristics are presented in **Table 1**.

PJI occurred in 148 (63%) cases of primary hip arthroplasty and in 88 (37%) cases of primary knee arthroplasty. Patients' mean age at time of infection was 68 years (SD 11.5 years), 147 cases (62%) were male and 89 cases (38%) were female.

### Prosthetic Joint Infection characteristics

The median interval between index procedure and diagnosis of infection was 20 days. On admission, 73% of the PJI patients had persistent wound leakage and 50% erythema at the affected joint. Of all PJI patients 49% had 2 or more clinical features at presentation. The predominant pathogens cultured from intraoperative samples during the first debridement were staphylococcus aureus (N= 107, 45%), coagulase negative staphylococcus (N=99, 42%), streptococcus sp. (N=32, 14%) and corynebacterium sp. (N=30, 13%). **Table 2.**

### Adherence to the protocol

Following the DAIR treatment, at the first debridement replacement of exchangeable parts significantly increased (p<0.01) from 24/53 cases (45%) in 2014, to 42/79 cases (53%) in 2015 and 73/104 cases (70%) in 2016. Over the years in less cases more than two times a debridement was performed (p<0.01) and an increase in the number of cultures obtained (≥6) from the affected prosthetic joint (p<0.001) **Table 3.**

### Outcome

Outcome following DAIR is detailed in **Table 4**. After 3 months follow-up, prosthesis retention was achieved in 215 (91%) cases and after 12 months follow-up in 205 (87%) cases. This latter percentage remained stable over the years (p>0.05). At the 12 months follow-up 24 (10%) of the patients required some form of surgery post-DAIR, for example a Girdlestone situation, amputation or one/two stage revision. Of our patients with PJI, 3% (N=7) died within one year after the primary surgery.

## Discussion

With this unique collaboration of nine hospitals in the South-East Netherlands we created a first regional registry, based on one combined treatment protocol for acute PJI of THA and TKA. We evaluated adherence to the treatment protocol. Moreover, this is the first study on diagnosis and treatment of PJI, which includes a large number of cases from different hospitals in the Netherlands using such a well-defined protocol.

The PJI incidence within the regional cohort was 1.12%, which is comparable to the range of 0.5-2% in literature. 2,3,20 The risk factors that are associated with developing PJI following total joint arthroplasty include male sex, age, higher body mass index (BMI), and the presence of comorbidities such as diabetes, cardiac history. These factors are also associated with a higher mortality risk, suggesting there would be an association between PJI and mortality. 21 Moreover, the mortality rate was 3% within one year after total joint arthroplasty infection, in line with literature (3-4%). 22,23 For comparison, the relative five-year survival rate for the most common cancers are 99% for prostate cancer, 89% for breast cancer, 64% for colorectal cancer 16% for lung and bronchial cancer and 91% for melanoma. 24 The high mortality rates related to PJI support the notion of a devastating complication and should be part of counseling of the patients 21,23.

Several guidelines have been developed to aid the challenging of diagnosis and treatment of PJIs. [e.g. 2,11] The regional protocol was based on these evidence-based statements in order to standardize PJI management. In line with our results, Armstrong et al. (2018) recently demonstrated that adherence to specific aspects of a PJI guideline varies considerably. They suggested that lack of awareness or not recognizing the importance of these aspects might be underlying issues for a not optimal protocol adherence. 12 The implementation of our regional treatment protocol for PJI involved regular discussion between the participating hospitals to get everybody on board, to clarify used definitions and to present analyses of the registered data. Over the course of the collaboration we found an increase in adherence to key-components of the treatment protocol. The better adherence was not yet reflected in better outcome.

### Limitations

The diagnosis of PJI is complex; multiple diagnostic tools are used in the attempt to correctly diagnose PJI. 20 Nowadays, the MSIS criteria are generally accepted worldwide and its use in research allowed for consistency in definition between studies. For the current analyses we used one of the major diagnostic criteria of MSIS; at least two phenotypically identical pathogens isolated from at least two separate tissues to identify true PJI cases. 2,4,18 In recent years, also numerous markers have been evaluated and described as minor criteria for PJI. However, as Parvizi et al. (2018) mentioned, while major criteria for infection are identical between the different definitions, the minor criteria differ and are less agreed upon. Publications in recent years have shown different weights (sensitivity and specificity) for the various tests (minor criteria) used. 18 Furthermore, PJI might be present if fewer criteria are met and even if all perioperative obtained cultures were negative 10. Unfortunately, as not all minor criteria were integrated in the online database, these data were not available to take into account to identify true PJI. In the current analyses, the definition of PJI was created using the most objective unbiased criteria that identified the strongest major diagnostic criteria. As a result there is no doubt about the accuracy of the PJI diagnosis in the identified cases.

Furthermore, the multicenter design of the study with incorporation of data from multiple centers in the regional PJI database may be caused variation in data interpretation. During the regional meetings we discussed this and reached consensus to minimize missing data. Such comparative data sharing improves the quality of care of PJI. The great variability of the way surgeons diagnose PJI, e.g. the interpretation of subjective assessments of tissue inflammation, is a major reason for underestimating the prosthetic joint infection. 17,18 Although the total number of primary THAs and TKAs performed in the participating hospitals only increased minimally, we noticed an increase in PJIs in our cohort over the three years evaluated. We believe this is rather a sign of better adherence to the protocol and better registration instead of a real increase of PJIs.

## Conclusion

Results of the regional cohort are in line with the available literature. Regional collaboration and regular feedback on registered data resulted in better adherence to the combined treatment protocol. Despite our attempts to improve PJI care, PJI remains a serious complication of THA and TKA with a significant mortality rate and great impact on the patients and health care. Since it is still not possible to determine a reliable incidence of PJI from the (Dutch) arthroplasty register, a specified PJI register is necessary to evaluate incidences and follow-up results. Such a register will enhance the collaboration between hospitals where experience and treatment results are routinely evaluated and discussed, to improve the quality of care around acute PJI of hip and knee arthroplasty.

## Figures and Tables

**Table 1:** Patient demographics, baseline clinical characteristics and prosthetic characteristics.

Variables	N	(%)
Joints Hip arthroplasty Knee arthroplasty Sex Male Female	148 88 147 89	(63%) (37%) (62%) (38%)
Age (years)* Body mass index (kg/m^2^)* Normal [18.5-24.9] Overweight [25-29.9] Obesity [30-34.9] Morbid obesity [≥ 35]	68 29.9 35 99 66 36	(± 11.5) (± 5.2) (15%) (42%) (28%) (15%)
Risk factors Diabetes mellitus Malignancy Others (e.g. cardiac history) Rheumatoid arthritis Smoking	15 15 55 14 30	(6%) (6%) (23%) (6%) (13%)

*presented as mean (±SD)

**Table 2:** Predominant organisms cultured from intraoperative samples during the first debridement.

Organism	N	(N%)
*Staphylococcus aureus*	107	(45%)
Coagulase negative staphylococcus	99	(42%)
*Corynebacterium* species	30	(13%)
*Streptococcus* species Haemolyticus group A Haemolyticus Group B Haemolyticus Group C Haemolyticus Group G *Enterococcus* species	32 2 3 2 8 33	(14%) (1%) (1%) (1%) (3%) (14%)

**Table 3:** Adherence to the regional protocol.

	2014		2015		2016		Total	
	N	(%)	N	(%)	N	(%)	N	(%)
**No. of debridements**								
1	30	(57%)	57	(72%)	63	(60%)	150	(64%)
2	13	(24%)	15	(19%)	38	(37%)	66	(28%)
≥3^**^	10	(19%)	7	(9%)	3	(3%)	20	(8%)
^*^Exchange of components								
Yes^**^	24	(45%)	42	(53%)	73	(70%)	139	(59%)
**No. of cultures**								
≥6^**^	22	(42%)	58	(73%)	73	(70%)	153	(65%)

*Results of 'Exchange of components' and 'No. of cultures' are based on the first debridement.** Significant difference across the years.

**Table 4:** Outcome following DAIR and complications.

	3 months	(%)	12 months	(%)
**Prosthesis retention**	215	(91%)	205	(87%)
**Reoperation** One-stage revision Two-stage revision Girdlestone Amputation Unknown^*^	17 6 3 4 1 3	(7%) (2.5%) (1.3%) (1.7%) (0.4%) (1.3%)	24 4 14 4 1 0	(10%) (1.7%) (5.9%) (1.7%) (0.4%) (0%)
**Mortality**	4	(2%)	7	(3%)

* In three cases the type of reoperation at three months was not defined.
